# Efficient 3D-CTF correction for cryo-electron tomography using NovaCTF improves subtomogram averaging resolution to 3.4 Å

**DOI:** 10.1016/j.jsb.2017.07.007

**Published:** 2017-09

**Authors:** Beata Turoňová, Florian K.M. Schur, William Wan, John A.G. Briggs

**Affiliations:** a Structural and Computational Biology Unit, European Molecular Biology Laboratory, Meyerhofstrasse 1, Heidelberg, Germany; b Structural Studies Division, MRC Laboratory of Molecular Biology, Francis Crick Avenue, Cambridge, UK

**Keywords:** Cryo-electron microscopy, Tomography, Contrast transfer function, Subtomogram averaging, Defocus, Reconstruction, Weighted back projection

## Abstract

Cryo-electron tomography (cryo-ET) allows cellular ultrastructures and macromolecular complexes to be imaged in three-dimensions in their native environments. Cryo-electron tomograms are reconstructed from projection images taken at defined tilt-angles. In order to recover high-resolution information from cryo-electron tomograms, it is necessary to measure and correct for the contrast transfer function (CTF) of the microscope. Most commonly, this is performed using protocols that approximate the sample as a two-dimensional (2D) plane. This approximation accounts for differences in defocus and therefore CTF across the tilted sample. It does not account for differences in defocus of objects at different heights within the sample; instead, a 3D approach is required. Currently available approaches for 3D-CTF correction are computationally expensive and have not been widely implemented. Here we simulate the benefits of 3D-CTF correction for high-resolution subtomogram averaging, and present a user-friendly, computationally-efficient 3D-CTF correction tool, NovaCTF, that is compatible with standard tomogram reconstruction workflows in IMOD. We validate the approach on synthetic data and test it using subtomogram averaging of real data. Consistent with our simulations, we find that 3D-CTF correction allows high-resolution structures to be obtained with much smaller subtomogram averaging datasets than are required using 2D-CTF. We also show that using equivalent dataset sizes, 3D-CTF correction can be used to obtain higher-resolution structures. We present a 3.4 Å resolution structure determined by subtomogram averaging.

## 1 Introduction

In cryo-ET, the 3D scattering potential of a vitrified specimen is reconstructed from a series of 2D projection images taken from different angles (a tilt-series) using a transmission electron microscope. The reconstructed volume (a tomogram) contains not only information about the structural features of an object of interest but also contextual information such as the location of the object, and its surrounding environment. If multiple instances of an object of interest are present within one tomogram or a set of tomograms, they can be computationally extracted to obtain a set of 3D subtomograms. The subtomograms can be aligned and averaged to obtain a high-resolution structure of the object. The reader is addressed to a number of reviews on subtomogram averaging (Förster and Hegerl, 2007, Förster et al., 2010, Briggs, 2013, Wan and Briggs, 2016).

The signal recorded in the 2D projection images is modulated by the contrast transfer function (CTF) of the microscope. The CTF is a sinusoidal function of spatial frequency whose shape depends on microscope parameters including the defocus at which the image was taken. The CTF causes periodic signal inversion at certain spatial frequency ranges and the complete loss of information at zero-crossings. Therefore, in order to recover structural information at frequencies higher than the first zero-crossing, CTF correction must be applied, and images collected at different defoci have to be combined.

CTF correction requires knowledge of the defocus of the image, which is typically obtained by fitting theoretical CTFs to the power spectrum of the image. While this task is routine during 2D cryo-electron microscopy (cryo-EM), it is more challenging in cryo-ET. This is because factors such as the low electron dose used during cryo-ET image acquisition, the typically thicker specimens, and tilting of the specimen (which increases apparent specimen thickness), result in tilt-series images having lower signal-to-noise ratios (SNR). This weaker signal results in less reliable CTF-fitting. Furthermore, tilted images have a defocus gradient perpendicular to the tilt axis, resulting in different parts of the image having different defocus values. These problems can be partially overcome by periodogram averaging of strips parallel to the tilt axis. The strip size is chosen so the defocus variation is minimal and the resulting defocus measurements can be then used to calculate the mean defocus and/or defocus gradient (Mindell and Grigorieff, 2003, Fernández et al., 2006, Xiong et al., 2009). Finally, in thick specimens, objects are located at different heights within the specimen, and therefore are imaged at different defoci – mixing of these signals produces an additional envelope function hindering CTF determination.

A further set of challenges is associated with correcting the CTF. Ideally, CTF correction should take into account the defocus gradient across the image of a tilted specimen, and also the defocus gradient through the thick sample: objects at different heights within the thick specimen are imaged at different defoci. In most implementations of CTF correction for cryo-ET, the defocus gradient across the image is taken into account (Fernández et al., 2006, Xiong et al., 2009, Zanetti et al., 2009). This is most commonly achieved by performing CTF correction on strips parallel to the tilt axis using the respective local defocus values (Fernández et al., 2006, Xiong et al., 2009). The locally corrected strips are then recombined and interpolated to generate a corrected image. This is the implementation of CTF correction that is used in the most popular tomogram reconstruction package, IMOD (Kremer et al., 1996, Xiong et al., 2009).

Dealing with the defocus gradient through the thick sample, often referred to as “3D-CTF correction”, is more difficult, and is not done in most CTF correction protocols. Some approaches have, however, been proposed and implemented. The problem is equivalent to that of reconstructing a large icosahedral virus in single-particle methods, taking into account the limited depth-of-field in the image (the top and bottom of the virus are at different defoci in the image). Jensen and Kornberg (2000) proposed a solution to this problem. They suggested to correct each image with multiple defocus values. They then proposed to divide the volume to be reconstructed into slices perpendicular to the direction of view for each image, and to back-project the information from the image corrected with the appropriate defocus value into each slice. Computationally very demanding at that time, the approach was only assessed using synthetic data. This solution was shown to be mathematically valid by Kazantsev et al. (2010). A different approach implements reconstruction in the form of direct Fourier inversion where the CTF is corrected directly within the reconstruction process (Voortman et al., 2012). Although the method showed promising results on synthetic data, the extensive computation times (in order of days) prevented this method from being widely used.

Recently Kunz and Frangakis (2017) proposed a GPU implementation of 3D-CTF correction related to the Jensen and Kornberg (2000) approach in combination with two different reconstruction techniques: Weighted Back Projection (WBP) (Bracewell and Riddle, 1967, Crowther et al., 1970, Gilbert, July 1972) and Simultaneous Algebraic Reconstruction Technique (SART) (Andersen and Kak, 1984). Rather than correcting input images, Kunz and Frangakis rotated the tomographic volume for each tilt, and corrected 2D slices within the tomographic volume. They were able to demonstrate small improvements at intermediate resolutions within reasonable computation time, showing that practical 3D-CTF correction can be feasible.

When subtomogram averaging is being performed, an alternative possibility to deal with the defocus variation is to perform CTF correction locally for each subtomogram, using defocus values dependent on the 3D positions of subtomograms within a tomogram. In the current subtomogram averaging implementation in RELION, Bharat and colleagues do this by performing CTF correction on subtomograms (extracted from uncorrected tomograms) using a Fourier volume representation of the CTF of the subtomogram (Bharat et al., 2015). This approach is inaccurate when the subtomogram is small. This is because Fourier components that are contributed by different tilt images, and that are therefore modulated by different CTFs, overlap in the Fourier transform of the extracted subtomogram. While they could have been independently corrected in the tilt images, they can no longer be independently CTF corrected in the extracted subtomogram. In an alternative approach, subtomograms are reconstructed from corresponding regions of tilt images that were corrected according to the 3D positions of the subtomograms (Chen and Förster, 2013, Zhang and Ren, 2012). This approach allows for proper CTF correction under conditions where the regions reconstructed are large enough to contain the structure of interest as well as its delocalized signal, but small enough not to contain a significant defocus gradient. The direct reconstruction of subtomograms is, however, a region of interest (ROI) reconstruction and may therefore also suffer from all associated artifacts (Turoňová et al., 2016).

In this work we propose, implement, and test an alternative, efficient implementation of the Kornberg and Jensen approach for cryo-ET and make this available in a software tool for 3D-CTF correction of tomograms that we call NovaCTF.

## 2 Methods and simulations

### 2.1 CTF definition

The CTF of a microscope can be described using following formula (Mindell and Grigorieff, 2003):

$$\mathrm{CTF}(f\text{,}\;\Delta z\text{,}\;\lambda \text{,}\;C_{s})=-w_{1}\sin(\pi \lambda f^{2}(\Delta z-0.5\lambda^{2}f^{2}C_{s}))-w_{2}\cos(\pi \lambda f^{2}(\Delta z-0.5\lambda^{2}f^{2}C_{s}))$$

where f denotes the spatial frequency, Δz the defocus,λ the electron wavelength, $C_{s}$ the spherical aberration of the microscope, and $w_{1}$ and $w_{2}$ denote relative phase and amplitude contrast (A):

$$w_{1}=\mathrm{sqrt}(1-A^{2})\text{,}$$

$$w_{2}=A\text{.}$$

The CTF causes inversion of signal at some spatial frequencies as well as complete loss of information at the zero-crossings. The aim of CTF correction is to restore the signal by reversing the sign of the inverted signal. This can be achieved by phase-flipping, where the sign is simply inverted; by multiplication with the CTF; or by Wiener filtering (Wiener, 1949), where CTF is divided by a frequency-dependent weighting function. For a detailed comparison of these three approaches we refer the reader to Downing and Glaeser (2008). We note that in a single image or single tomogram, the signal at the zero-crossings cannot be recovered and the image or tomogram is not strictly corrected. The only way to restore signal at zero-crossings is to combine information from particles imaged under different defoci, for example by subtomogram averaging. For simplicity we refer to phase-flipped or CTF-multiplied images and tomograms as “corrected”.

### 2.2 Outline of method for 3D-CTF

**Fig. 1:**
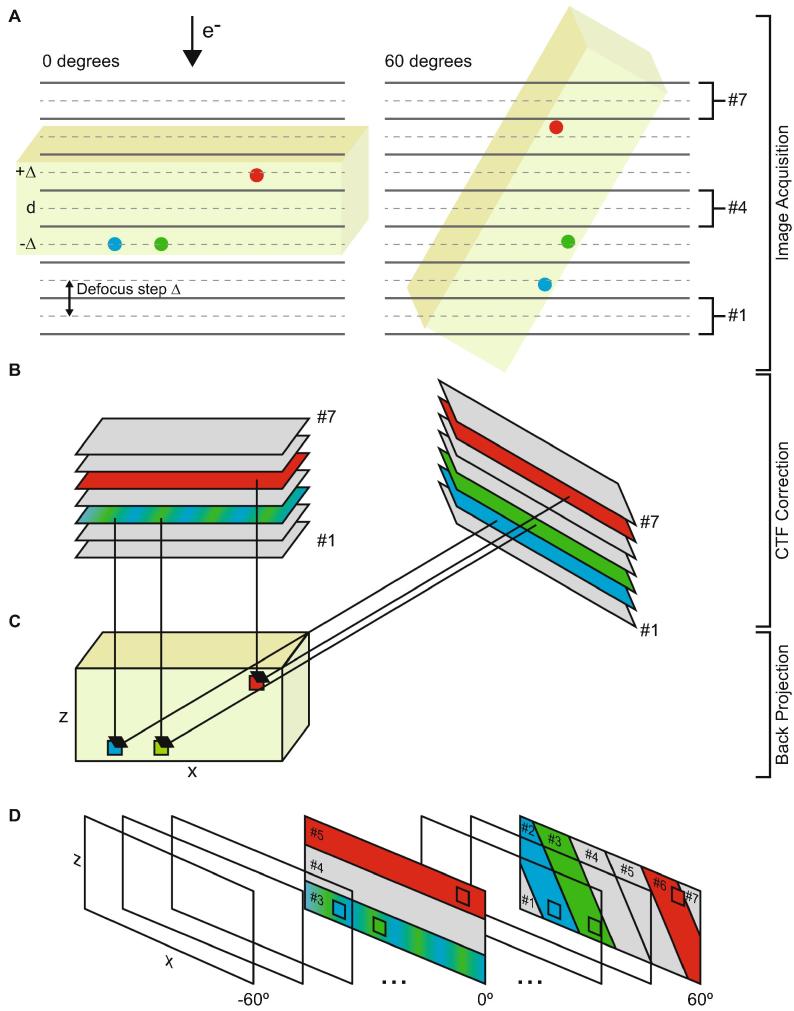
A. During imaging of the sample (green slab) at 0 degree tilt, objects at the positions marked by the blue and green circles have a defocus of d - Δ, while one at the position of the red circle has a defocus of d + Δ. When imaged at 60 degree tilt, the blue position has a defocus d - 2Δ, the green position has defocus of d - Δ, and the red position d + 2Δ. These objects are projected into an image. B. In this example, a defocus step of Δ is used, so each tilt image is CTF corrected seven times in steps of Δ, to give images #1 (d − 3Δ) to #7 (d + 3Δ). C. During back projection to generate the reconstruction volume (green cuboid), the blue voxel, corresponding to the position of the blue circle in A, will be reconstructed using image #3 at 0 degrees, and image #2 at 60 degrees; the green voxel, corresponding to the position of the green circle in A, will be reconstructed using image #3 at 0 degrees, and image #3 at 60 degrees; while the red voxel will be reconstructed using image #5 at 0 degrees, and image #6 at 60 degrees. Note that the reconstruction volume should be positioned such that its center has defocus d. This will be approximately the case if the center of mass corresponds to the center of the tomogram. If this is not the case, an additional defocus shift can be applied. D. Cartoon illustrating the defocus array. The defocus array is three-dimensional, with axes corresponding to x, z and tilt angle, and stores indices of images that should be used to reconstruct each voxel.

Here we present and test an implementation of the approach proposed by Jensen and Kornberg (2000) adapted for cryo-ET (Fig. 1). Our implementation is different to that proposed by Kunz and Frangakis (2017), and avoids rotating the reconstruction volume for each tilt angle, eliminating the additional interpolation errors and computational cost of this volume rotation.

Correct implementation of the Jensen and Kornberg approach requires that during the reconstruction of the tomogram by back-projection, each voxel is calculated from tilt images that were CTF-corrected with defocus values corresponding to the position (given by x and z coordinates) of that voxel at each tilt (see Fig. 1). To achieve this, each image in the tilt-series is CTF-corrected multiple times with different defocus value. The number of different CTF corrections performed per image depends upon how finely the defocus gradient should be sampled, and becomes a user-defined variable (*defocus step*). Prior to the reconstruction, we precompute a defocus array which stores the indices of the CTF-corrected tilt images that should be used to reconstruct each voxel. Since we orient the reconstruction volume with the tilt axis parallel to the y-axis (zero x-tilt in IMOD, Mastronarde, 2006), we only need to compute the indices for a single 2D volume slice in the xz-plane for each tilt image. The defocus array is thus three-dimensional, with axes corresponding to x, z and tilt angle (see Fig. 1D). The reconstruction itself is then performed using WBP in a manner equivalent to the implementation in IMOD (Kremer et al., 1996, Mastronarde, 1997). In the IMOD implementation, each voxel is projected onto each tilt image to find its position within that tilt image and simple linear interpolation is used to obtain the value at that position. The values from each tilt image are then added up to obtain a final voxel value. The difference in our approach is that for each voxel we use the precomputed defocus array to determine which tilt images were CTF-corrected with the proper defocus value, and compute the values from those images.

By taking into account the defocus gradient through the thick specimen, this approach also takes into account the defocus gradient across the tilted image. Each CTF correction performed on an image in the tilt-series is therefore a simple CTF correction on the full image. This correction is significantly faster than strip-based approaches and avoids possible interpolation errors. Unlike strip-based approaches it can also be efficiently performed prior to tilt-series alignment. In our implementation, since CTF correction is done as a preprocessing step, we can easily exchange between different CTF correction algorithms without the need to adapt the reconstruction process itself.

### 2.3 Implementation

The reconstruction procedure from IMOD was rewritten from FORTRAN into C++ and was extended to allow reading from multiple tilt images based on the precomputed defocus array. To decrease memory requirements, the original FORTRAN implementation splits the reconstructed volume as well as the tilt-series along the y dimension and keeps in memory only as many xz slices and corresponding rows from tilt-series as can be fit. After the slices are reconstructed they are written out and next batch is loaded. In our implementation we keep only one xz slice, and one row from each CTF-corrected tilt image in memory.

The workflow for 3D-CTF correction was designed to fit within the standard workflow of IMOD eTomo, including parameter setup as well as the orientations of tilt-series and the final tomogram. Instead of using the strip-based correction (Xiong et al., 2009) provided by IMOD, we provide our own implementation of a simple CTF correction done by either phase-flipping or multiplication, and which can take account of astigmatism. Instead of performing row-wise radial filtering within the weighted back projection reconstruction procedure (as done in IMOD), we filter the CTF-corrected images using the same radial filter prior to the reconstruction. This change allows filtering to be parallelized on the level of tilt-series without altering the outcome of the reconstruction.

Source code for NovaCTF is distributed under an LGPL license and can be downloaded including installation scripts from: https://www.embl.de/research/units/scb/briggs/services/index.php or from https://github.com/turonova/novaCTF.

## 3 Results

### 3.1 Simulations

We first estimated the improvement in signal recovery that could be expected through use of 3D-CTF correction. To do this we simulated the attenuation of signal caused by errors in CTF correction and determination for both 2D-CTF and 3D-CTF. Simulations were performed in a manner similar to those performed in Schur et al. (2013). We assume a large number of subtomograms, distributed through multiple tomograms collected at different defoci, as is the case in a typical subtomogram averaging experiment. For each subtomogram position we generate the corresponding CTF, which we phase-flip either correctly (the “true phase-flipped CTF”), or based on node positions calculated using defocus values with a defined defocus error (the “experimental phase-flipped CTF”). The defocus error is the difference between the z-position of the subtomogram and the z-position for which the defocus is defined. We calculate the sum of all the “experimental phase-flipped CTFs” and divide it by the sum of the “true phase-flipped CTFs” to obtain a curve that describes the attenuation due to the defocus error. For 2D-CTF correction, the defocus is defined at the center of the tomogram and the defocus error is the distance from the center of the tomogram to the center of the subtomogram. For 3D-CTF correction, the defocus is defined at the center of the defocus step and the defocus error is the distance of the subtomogram from this position. We note that these simulations approximate low-tilt-angle images from which most high-resolution information is obtained. At higher-tilts, there is no change in attenuation of information when 3D-CTF correction is applied, but when 2D-CTF correction is applied the defocus error increases by 1/cos(tilt angle) leading to increased loss of signal. We can also incorporate additional error for imprecise defocus determination. As we show below, these simulations can also be used to determine an appropriate defocus step.

**Fig. 2:**
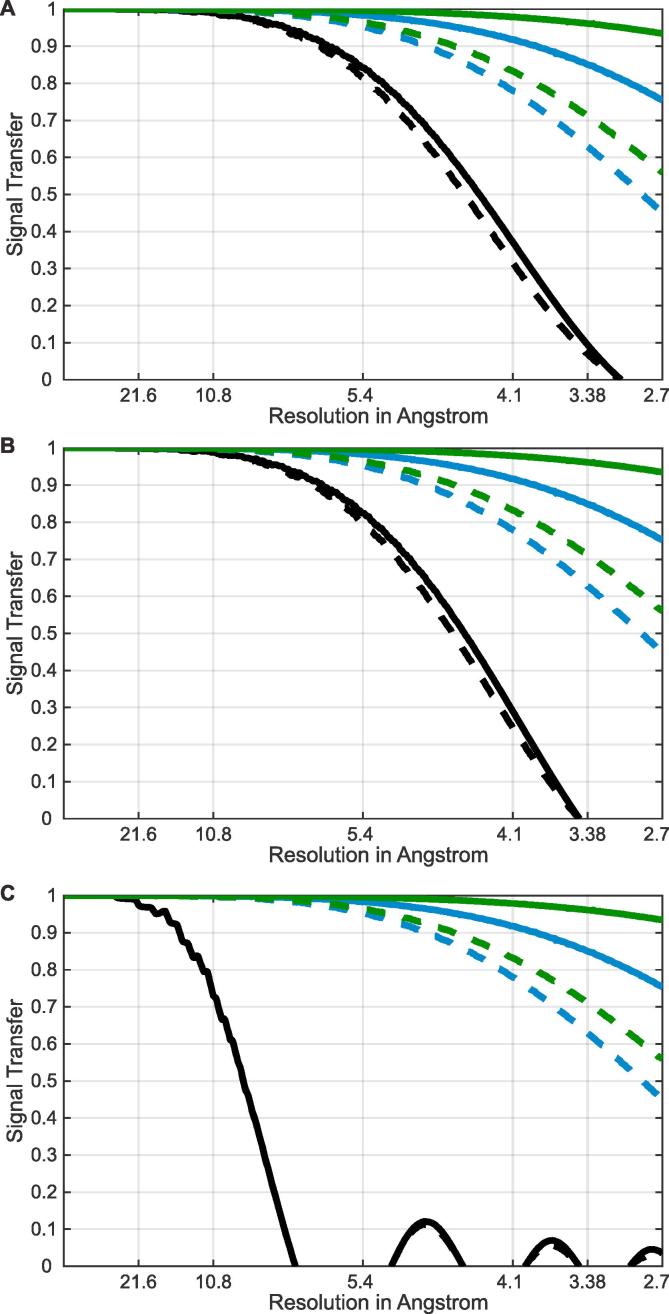
Simulation of the signal attenuation caused by CTF modulation. A. Simulations are based on the experimental dataset described here using CTF correction by phase-flipping. The average thickness of tomograms is around 160 nm. Solid lines assume there is no error in defocus determination, dashed lines assume a normally distributed error in defocus determination with 0 nm mean and 12 nm standard deviation. Black lines are for 2D-CTF correction, blue lines are for 3D-CTF correction with a defocus step of 30 nm, green lines are for 3D-CTF correction with a defocus step of 15 nm. 3D-CTF correction substantially improves signal recovery when compared to 2D-CTF correction, especially at resolutions beyond 5 Å. B. The same simulations using the same number of subtomograms and defoci but with subtomograms uniformly distributed within 100 nm thickness tomograms. The benefits of 3D-CTF correction can be observed only at high-resolution. C. The same simulations as in B, with subtomograms uniformly distributed within 500 nm thickness tomograms. The 3D-CTF correction significantly improves the chances of obtaining structures with resolution higher than 10 Å.

Here, we performed these simulations for data collected under our typical experimental conditions. We therefore used the number, positions and defoci of the subtomograms in the experimental dataset used below (Schur et al., 2016) for testing to calculate the error distribution. We calculated the attenuation curve for 2D-CTF, for 3D-CTF with a 30 nm defocus step, and for 3D-CTF with a 15 nm defocus step. We also calculated the same attenuation curves assuming an additional normally-distributed error with standard deviation of 12 nm to take into account imprecision in defocus determination and imprecision in determining the position within the tomogram corresponding to the measured defocus. The results of these simulations are shown in Fig. 2A. At resolutions worse than 1 nm, there was no benefit in using 3D-CTF when compared to 2D-CTF for this dataset. There were substantial gains at high-resolution (beyond 5 Å): approximately 3-fold more signal could be recovered at 4.1 Å using 3D-CTF correction than using 2D-CTF correction. A smaller defocus step slightly increased the recovery of higher-resolution information, but increased processing time as outlined below. Thus, in resolution ranges currently achieved by subtomogram averaging, there is little benefit obtained by using a defocus step below 15 nm.

To simulate the benefits of 3D-CTF correction for tomograms of different thickness, we repeated the simulation using the same number of subtomograms and defoci as in the experimental dataset but with the subtomograms distributed uniformly within 100 nm and 500 nm thick tomograms. The results, shown in in Fig. 2B and C, respectively, show that 3D-CTF correction can improve the resolution even for thin specimens and is essential to achieve high-resolution structures in the case of thick specimens.

### 3.2 Validation using synthetic data

To allow the benefits of 3D-CTF correction to be properly assessed, for both validation and testing we perform parallel experiments using IMOD’s ctfphaseflip and WBP. Since NovaCTF uses the same back projection implementation as IMOD, the impact of 3D-CTF correction can be assessed without influence from other changes in the reconstruction pipeline.

**Fig. 3:**
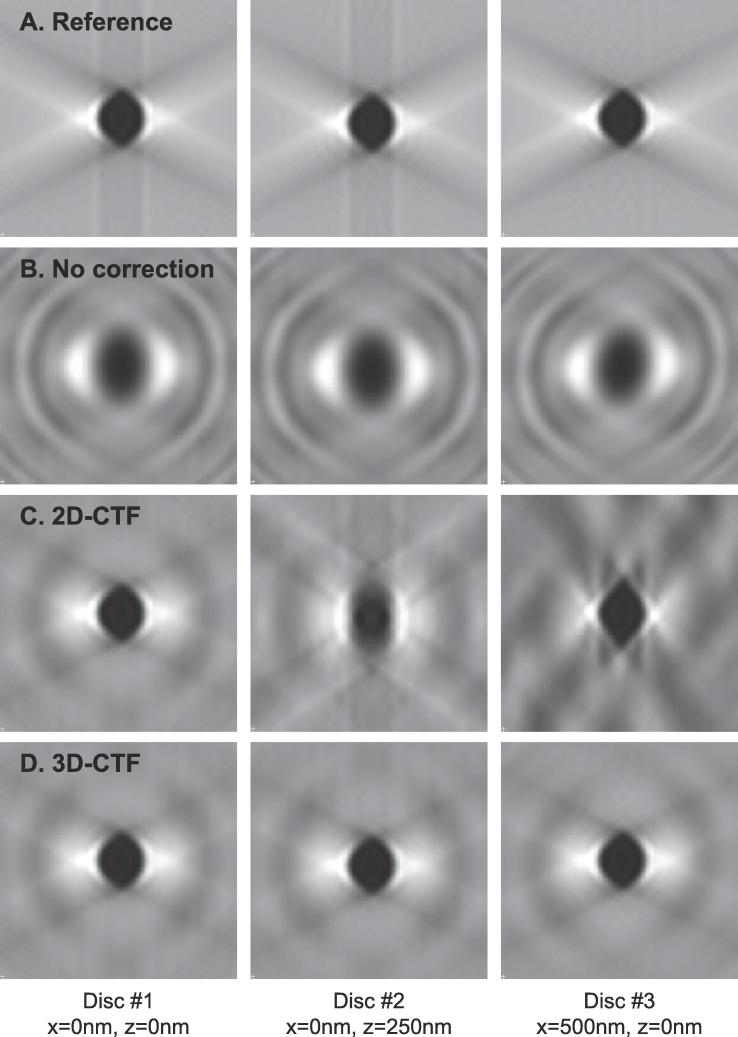
Validation using a synthetic dataset. The synthetic dataset was a volume of 1065 nm × 1065 nm (in xz dimension) containing three discs with the same intensity and diameter 2.6 nm. The positions of the discs are given from the center of the tomogram (x = 0 nm, z = 0 nm). A. The results of reconstruction from a tilt-series simulated for a microscope with no CTF distortion. The only artifacts present are those caused by the missing wedge or the reconstruction protocol. B. The results of reconstruction from a CTF-modulated tilt-series with no CTF correction applied during the reconstruction. C. The results of reconstruction using the 2D-CTF correction approach implemented in IMOD’s ctfphaseflip. D. The results of reconstruction using 3D-CTF reconstruction (phase-flipping and slice thickness 60 nm). While the 2D-CTF approach only recovers the central disc correctly, 3D-CTF manages to recover the correct structure regardless of a disc’s position within the tomogram.

We performed validation experiments using the synthetic dataset presented in Voortman et al. (2012): this is a 2D xz slice of 1065 nm × 1065 nm containing three discs – disc #1 at coordinates x = 0, z = 0 (corresponding to the center of the volume), disc #2 at x = 0, z = 250 nm, and disc #3 at x = 500 nm, z = 0. Each disc has diameter 2.6 nm. We used the scripts from Voortman et al. (2012) to simulate the input tilt-series from the volume, modifying the parameters for CTF distortion to better match our experimental conditions: pixel size 2.6 Å, spherical aberration 2.7 mm, defocus −4000 nm and 300 keV. The tilt range was from −60 to +60 degrees with angular step size 3 degrees resulting in 41 images in one tilt-series. Two tilt-series were generated: one with simulated CTF and one without (i.e. simple projections of the volume without any CTF distortions), to distinguish between artifacts caused by CTF and those caused by the missing wedge. We reconstructed the volume using IMOD either in the absence of any CTF correction, or using IMOD's ctfphaseflip implementation. We then reconstructed the volume using our 3D-CTF approach with a defocus step of 60 nm. To allow a direct comparison with the IMOD results we corrected the CTF by phase-flipping. The results are shown in Fig. 3. While 2D-CTF correction only recovers the central disc correctly, our 3D-CTF approach recovers the structure of all discs, regardless of their position within the tomogram.

### 3.3 Testing using experimental data

We next tested our method by applying it to an experimental dataset from Schur et al. (2016) from which the structure of the immature HIV-1 CA-SP1 lattice was determined. The dataset consists of 43 tomograms (3710 × 3710 × ∼1200 voxels) containing HIV-1 dMACANC VLPs from which ∼130,000 subtomograms of size 192^3^ were aligned and averaged to generate the final structure. In the original analysis, CTF measurement was performed using CTFFIND4 (Rohou and Grigorieff, 2015) (parameters: box size 512, min resolution 30.0 Å, max resolution 5.0 Å, minimal defocus 500 nm, maximal defocus 5500 nm), CTF correction was performed using ctfphaseflip from IMOD, and tomograms were reconstructed using IMOD’s WBP. The final structure had a resolution of 3.9 Å.

We reprocessed this data under a range of different conditions (see below). In each case we determined the resolution by averaging 5 phase-randomized FSC curves computed according to Chen et al. (2013). For 3D-CTF correction, we used a defocus step of 15 nm, resulting in approximately 33 corrected images for each image in the tilt-series. All other relevant parameters were identical to those used in the original work.

**Fig. 4:**
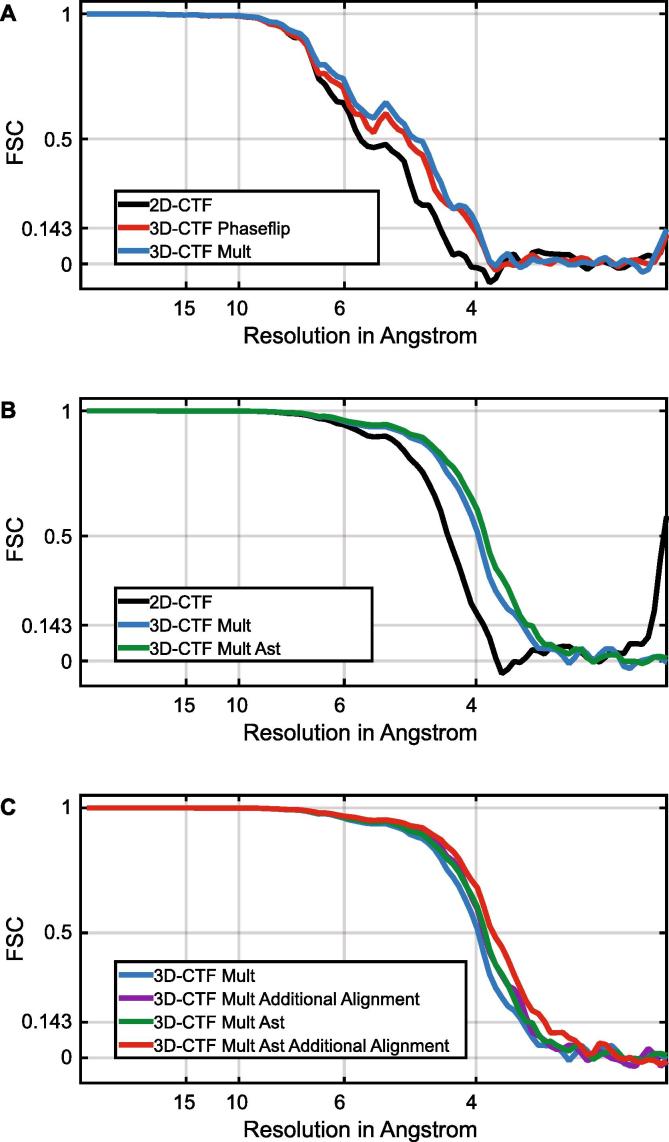
FSC curves obtained by reconstructing experimental data under different conditions. A. FSC curves for averages obtained from 5 tomograms using the alignment information (shifts and rotations) from the original analysis described in Schur et al. (2016). 2D-CTF correction (black) was performed using IMOD’s ctfphaseflip. 3D-CTF correction was performed using phase-flip (red) or multiplication (blue). The 3D-CTF curves shows better correlation at higher frequencies, with multiplication slightly outperforming phase-flipping. B. FSC curves for averages obtained using 43 tomograms (i.e. the whole original dataset). The 2D-CTF curve (black) corresponds to the original data from Schur et al. (2016). For 3D-CTF, the structure was obtained by averaging the subtomograms using the shifts and rotations found in Schur et al. (2016). The 3D-CTF using multiplication (blue) significantly improved the resolution of the structure. An additional improvement was achieved by considering astigmatism during multiplication (green). C. FSC curves for averages obtained as in B, and also for averages obtained after one further round of alignment using the 3D-CTF corrected average as a reference, either without (purple), or with (red) astigmatism correction.

Initially we selected 5 tomograms from the dataset, evenly covering the defocus range from the whole dataset. We generated the tomograms using the strip-based 2D-CTF applied in IMOD as in the original manuscript, or using our 3D-CTF correction applying phase-flipping. From each set of tomograms we then extracted subtomograms and averaged them using the alignment information (translations and rotations) from the original analysis in Schur et al. (2016). The results are shown in Fig. 4A. The measured FSC curves clearly show an improvement in resolution when using the 3D-CTF (4.0 Å), over 2D-CTF (4.4 Å). We also generated the tomograms using our 3D-CTF approach but instead of phase-flipping, we multiplied by the CTF, and averaged the subtomograms as above. The resulting average is modulated by the CTF squared, and is therefore reweighted by dividing by the sum of the squared CTFs corresponding to all subtomograms. Although the resolution at the “headline” 0.143 criterion does not improve, there is a clear movement of the FSC curve to the right: multiplication outperforms phase-flipping. In all subsequent experiments we therefore applied CTF multiplication.

We next compared the performance of 2D-CTF with the 3D-CTF (applying CTF multiplication) using the full dataset of 43 tomograms. Again we used the alignment information obtained in the original work. The structure from 3D-CTF corrected data showed an improvement in resolution of 0.3 Å (to 3.6 Å) compared to the 2D-CTF approach (Fig. 4B). We next assessed whether a further improvement in resolution could be obtained if we also considered astigmatism during CTF measurement and correction. Image astigmatism was measured using CTFFIND4, and CTF multiplication was performed with an astigmatic CTF (Mindell and Grigorieff, 2003, Henderson et al., 1986). When we regenerated the structure, the resolution improved further to 3.5 Å.

So far, all averages were generated using the rotations and shifts calculated in the original study using 2D-CTF correction. We next assessed whether a better subtomogram alignment could be obtained by using the improved structure obtained with 3D-CTF correction as a reference. We ran one additional iteration of subtomogram alignment using the 3D-CTF corrected structure as a reference, applying subtomogram alignment parameters identical to those used in the last iteration from the original analysis. We did this both with and without considering astigmatism. In both cases the resolution of the structure improved (Fig. 4C), to 3.5 Å without astigmatism correction, and to 3.4 Å considering astigmatism correction.

**Fig. 5:**
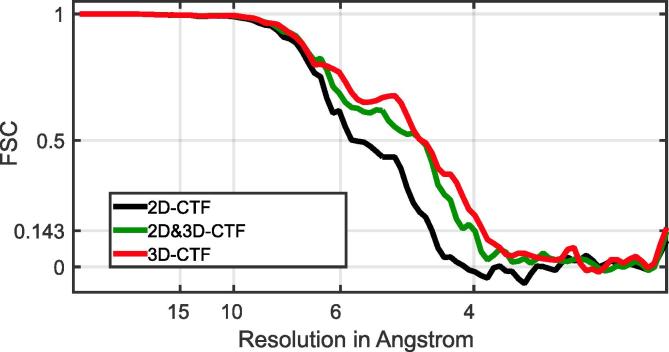
Comparison of FSC curves for structures obtained by performing the whole subtomogram averaging pipeline on data from 5 tomograms only. The results obtained using 2D-CTF correction as implemented in IMOD are shown in black. When the shifts and rotations from this alignment were used, but the final average was calculated using subtomograms from 3D-CTF corrected tomograms we obtained the green curve. When the complete pipeline was performed on 3D-CTF-corrected tomograms we obtained the red curve.

We then considered again the smaller dataset of 5 tomograms and performed the complete subtomogram averaging workflow from scratch, without making use of any of the alignment parameters determined during the original analysis. First, as a control, we used the 2D strip-based approach, obtaining a structure with resolution of 4.5 Å. Second, we used rotations and shifts calculated from this control experiment, but generated the final average from the 3D-CTF corrected tomograms, which improved the resolution to 4.0 Å. Third we performed the whole workflow on 3D-CTF corrected tomograms from scratch, obtaining a resolution of 3.9 Å (Fig. 5). Although the initial number of subtomograms was the same for both datasets, the number of subtomograms used in the final average was slightly larger for the 3D-CTF approach (11,314, corresponding to 67,884 asymmetric units), than for the 2D-CTF approach (9901 subtomograms, corresponding to 59,406 asymmetric units). This can partially be attributed to using the 3D-CTF workflow from the very beginning of the processing pipeline: the subtomograms are better aligned at earlier stages of processing, and therefore fewer misaligned subtomograms are excluded from later stages of processing. For a detailed description of the workflow we refer the reader to Schur et al. (2016).

**Fig. 6:**
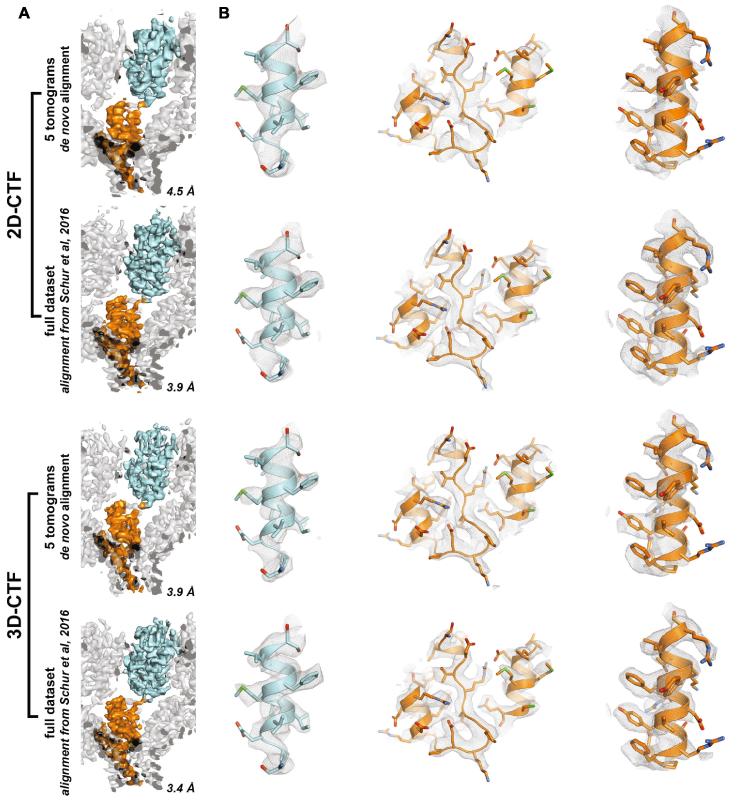
Comparison of structural features in 2D-CTF and 3D-CTF corrected structures of HIV-1 CA-SP1. To allow easy comparison of the structures, similar views to those illustrated in Schur et al. (2016) have been chosen. A. Overview of the HIV-1 CA-SP1 structure. One CA-SP1 monomer is always shown with the CA-N-terminal domain and CA-C-terminal domain in cyan and orange, respectively. The resolution for each structure is annotated in the bottom right. B. For each structure three selected regions from the CA-SP1 monomer are shown in a close-up view.

These experiments show that the subtomogram alignment performs better on 3D-CTF corrected tomograms. The 3.9 Å structure from 5 tomograms is noisier than the original 3.9 Å structure obtained from the whole dataset of 43 tomograms, and this is reflected in the smaller area underneath the FSC curve (compare Figs. 4C and 5). The level of detail in the structure is, however, consistent with the measured resolution (Fig. 6). Application of a 3D-CTF approach therefore allows a similar resolution to be achieved with a dataset roughly 10% of the size used in the original work of Schur et al. (2016). This reduction in the amount of data required is approximately consistent with the simulated attenuation curves shown in Fig. 2.

**Fig. 7:**
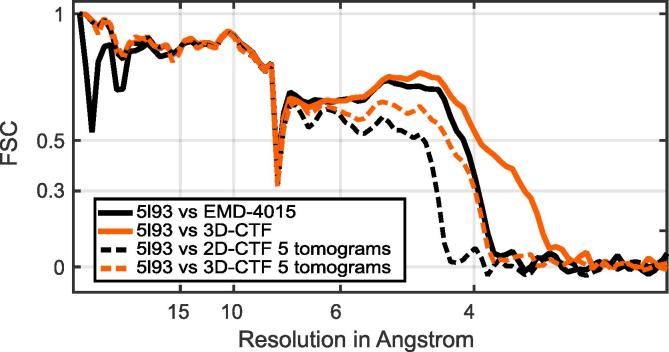
FSCs between the structures obtained and pdb 5l93. The structures obtained here and shown in Fig. 6 were compared to pdb 5l93 from Schur et al. (2016). 5l93 was generated by refining into EMD-4015 (the 2D-CTF corrected structure at 3.9 Å). The 3D-CTF corrected structures show better correlation with the structural model than the 2D-CTF corrected structures. We note that the model obtained by refining into the 3.9 Å structure is reliable to higher resolution.

As a further test of the resolution obtained using 3D-CTF, we calculated FSCs between the structures obtained and the structural model (pdb 5l93) generated by fitting into the previously published 3.9 Å structure EMD-4015 (Schur et al., 2016) (Fig. 7).

### 3.4 Time, memory and storage requirements

Our implementation of 3D-CTF requires that each image is CTF corrected multiple times. Nevertheless, the correction is less computationally demanding than strip-based approaches, and can be easily parallelized. For the experimental dataset we provide an overview of the computational times (Table 1), and memory/storage requirements (Table 2). We find that there is no substantial increase in processing time when using smaller defocus steps. There is an increase in storage requirements for smaller defocus steps, but these are only on the scale of the storage space required for one tomogram, and therefore not limiting. There is a doubling of total correction/reconstruction time compared to using IMOD but these steps can be parallelized, and the overall processing time remains very small compared to other steps in the cryo-ET data collection and subtomogram averaging workflow.

**Table 1:** Comparison of computation times for CTF correction and reconstruction using different approaches. The size of one tilt image is 3710 × 3710 pixels, and a tilt-series contains on average 41 tilt images. The reconstructed tomogram has dimensions 3710 × 3710 × 1200 voxels. In our test dataset, a defocus step of 15 nm results on average in 33 corrections per tilt image. Times were measured on a Xeon E3-1231V3, 3.5 GHz, 32 GB RAM, on a single CPU without any parallelization. The 2D strip-based approach performed using IMOD’s ctfphaseflip is slower than the simple angle-independent CTF correction used in NovaCTF for a single CTF correction. The longer total running times for NovaCTF are mainly caused by performing multiple CTF corrections per tilt image, and a longer reconstruction time due to different memory handling of support structures for 3D-CTF correction. The total computation time remains small compared to other steps in the subtomogram averaging workflow.

Method	CTF Correction	Reconstruction	Total time
IMOD	19 m 36 s	31 m 57 s	51 m 33 s
NovaCTF – 1 correction per tilt image	01 m 56 s	1 h 0 m 03 s	1 h 1 m 59 s
NovaCTF – 11 corrections per tilt image	21 m 16 s	1 h 0 m 11 s	1 h 21 m 27 s
NovaCTF – 33 corrections per tilt image	1 h 03 m 48 s	1 h 0 m 43 s	2 h 04 m 31 s

**Table 2:** The memory and storage requirements for CTF correction and tomogram reconstruction using NovaCTF. The size of one tilt image is 3710 × 3710 pixels, and a tilt-series contains on average 41 tilt images. The reconstructed tomogram has dimensions 3710 × 3710 × 1200 voxels. The total memory needed by the reconstruction consists mostly of support structures such as the defocus array, while the volume slice and rows of corrected tilt images require only a small portion of the total memory. As a consequence, the memory requirements do not increase significantly with decreasing defocus step. There is an increase in the amount of temporary storage required during reconstruction when smaller defocus steps are used, but these requirements are not in any way limiting on typical computer systems.

	Total memory (RAM)	Temporary storage
NovaCTF – 1 correction per tilt image	734 MB	68.2 GB
NovaCTF – 11 correction per tilt image	740 MB	90.5 GB
NovaCTF – 33 correction per tilt image	753 MB	139.6 GB

## 4 Summary

Here we have presented a computationally efficient 3D-CTF correction protocol for cryo-electron tomography that fits within the canonical IMOD eTomo pipeline, and which overcomes the practical and theoretical limitations of other current implementations. We compared our approach to ctfphaseflip, the most widely used implementation of strip-based 2D-CTF correction for tomographic data, and demonstrated clear improvements in reconstruction quality using both synthetic and experimental data. Our tests suggest that the higher signal quality in tomograms reconstructed with 3D-CTF correction allows high-resolution reconstructions to be obtained using much smaller datasets than those required with 2D-CTF. We were able to obtain a structure at the same resolution as that presented in Schur et al. (2016) using only 10% of the original data size. When equivalently sized datasets are used, 3D-CTF allows higher-resolution structures to be generated.

We conclude that 3D-CTF correction allows superior signal recovery to 2D-CTF correction, and improves the results of high-resolution subtomogram averaging. Because there is little additional computational cost, we expect 3D-CTF correction to replace 2D-CTF as in the standard protocol for cryo-ET and subtomogram averaging.

The 3.4 Å structure of the HIV-1 dMACANC VLPs has been deposited in the EMDB with accession number EMD-3782. Source code for NovaCTF is distributed under an LGPL license and can be downloaded including installation scripts from: https://www.embl.de/research/units/scb/briggs/services/index.php or from https://github.com/turonova/novaCTF.
